# Mixture of Polyphenols and Anthocyanins from *Vaccinium uliginosum L.* Alleviates DNCB-Induced Atopic Dermatitis in NC/Nga Mice

**DOI:** 10.1155/2012/461989

**Published:** 2012-10-23

**Authors:** Min Ju Kim, Se-Young Choung

**Affiliations:** Department of Preventive Pharmacy and Toxicology, College of Pharmacy, Kyung Hee University, 1 Hoegi, Seoul 130-701, Republic of Korea

## Abstract

*Vaccinium uliginosum L*. (VU) possesses various biological properties, such as antioxidant and protective effects against VU-induced skin photoaging. The purpose of this study is to evaluate the effects of oral administration of a mixture of polyphenols and anthocyanins derived from VU on 2,4-dinitrochlorobenzene- (DNCB-) induced atopic dermatitis (AD) in NC/Nga mice. We assessed anti-AD effects in NC/Nga murine model for a period of 9 weeks. Oral administration of the mixture significantly alleviated the AD-like skin symptoms and clinical signs including ear thickness and scratching behaviors. Orally administrated mixture reduced the level of IgE and IgG1, whereas it increased the level of IgG2a in a dose-dependent manner. The calculated IgG1/IgG2a ratio for each mouse revealed that the mixture derived from VU also significantly reduced the Th2/Th1 ratio, IL-4 and IL-13 (as Th2 cytokines), IFN-**γ**, and IL-12 (as a Th1 cytokine) in spleens. In addition, it significantly decreased gene expression, such as IL-4, IL-5, CCR3, eotaxin-1, IL- 12, IFN-**γ**, MCP-1, and IL-17, in AD-like lesions and suppressed Th17. Histological analyses revealed that the epidermis thickness and number of inflammatory cells were significantly reduced. In conclusion, oral administration of the mixture in the DNCB-induced AD is confirmed to improve AD disease in mice.

## 1. Introduction 

Atopic dermatitis (AD) is a chronic and relapsing inflammatory skin disease relying on the interplay of environmental, immunological, and genetic factors [1]. The incidence of AD is continuously increasing worldwide, with a prevalence rate of approximately 10–20%, and is more common among infants and children. AD is a multifactorial skin disease, with complex interactions of immune innate and adaptive immune responses based on a strong genetic predisposition and triggered by environmental factors [2]. *Vaccinium uliginosum L*. (VU), known as bog bilberry, has been reported to have antioxidant and protective effects against VU-induced skin photoaging [3, 4]. A previous study showed that the berries have an anti-inflammatory effect [5]. We investigate the effects of polyphenols and anthocyanins derived from VU. In AD skin, scratching induces the production of cytokines and chemokines and upregulates the expression of adhesion molecules. These processes are followed by the infiltration of skin lesions by lymphocytes, mast cells, eosinophils, and neutrophils. AD is a biphasic inflammatory skin disease that can be considered to have two distinct phases [6]. In the acute phase, AD skin lesions predominantly secrete the Th2 cytokines IL-4, IL-5, and IL-13, whereas in the chronic phase, Th1 cells secrete IFN-*γ* [7, 8]. AD is provoked by Th1/Th2 immune responses [9]. The imbalance of Th1 and Th2 immune responses plays an important role in the development of AD [10, 11]. The inflammatory infiltrate of AD predominantly consists of dendritic cells (DCs) and memory CD4+ T cells. Essentially, all T cells infiltrating the skin lesions express the cutaneous lymphocyte-associated antigen (CLA). The recent discovery of a distinct T helper subset, referred to as Th17 cells, based in their IL-17 production has led to the transformation of the Th1/Th2 paradigm of immunity into a novel viewpoint [12]. We also measure the Th1, Th2, and Th17 cytokines to discover the efficacy of the polyphenol and anthocyanin mixture. The highest percentage of IL-17-producing cells was found in AD patients. These IL-17 activities could promote tissue fibrosis and chronicity of the inflammatory process [13]. It is well known that the pathogenesis of AD shows related but distinct properties of IgE-mediated hypersensitivity, reflecting the complex involvement of environmental, genetic, and nonimmunological factors [14, 15]. The initial mechanisms that induce skin inflammation in AD patients are unknown [16]. Topical steroids, emollients, and oral antihistamines are used as the first-line therapy for AD; however, many patients worry about the long-term use of these agents [17]. Therefore, there is a need for a new reagent that has minimal side effects. The purpose of our study is to show the effect of mixture fraction in NC/Nga mice treated with DNCB to induce AD.

*Vaccinium uliginosum L*. (VU) possesses various biological properties, such as antioxidant and protective effects against VU-induced skin photoaging. The purpose of this study is to evaluate the effects of oral administration of a mixture of polyphenols and anthocyanins derived from VU on 2,4-dinitrochlorobenzene- (DNCB-) induced atopic dermatitis (AD) in NC/Nga mice. We assessed anti-AD effects in NC/Nga murine model for a period of 9 weeks. Oral administration of the mixture significantly alleviated the AD-like skin symptoms and clinical signs including ear thickness and scratching behaviors. Orally administrated mixture reduced the level of IgE and IgG1, whereas it increased the level of IgG2a in a dose-dependent manner. The calculated IgG1/IgG2a ratio for each mouse revealed that the mixture derived from VU also significantly reduced the Th2/Th1 ratio, IL-4 and IL-13 (as Th2 cytokines), IFN-*γ*, and IL-12 (as a Th1 cytokine) in spleens. In addition, it significantly decreased gene expression, such as IL-4, IL-5, CCR3, eotaxin-1, IL- 12, IFN-*γ*, MCP-1, and IL-17, in AD-like lesions and suppressed Th17. Histological analyses revealed that the epidermis thickness and number of inflammatory cells were significantly reduced. In conclusion, oral administration of the mixture in the DNCB-induced AD is confirmed to improve AD disease in mice.

## 2. Materials and Methods

### 2.1. Preparation of *Vaccinium uliginosum L*. Fractions

*Vaccinium uliginosum L*. (VU) is a plant species of *Vaccinium* which is a genus of shrubs or dwarf shrubs in the plant family Ericaceae that inhabits North Korea, Europe, and America. VU is known to contain organic acids, vitamins, glycosides, and anthocyanins and has been reported to have antioxidant activity [18, 19]. The aqueous solution was evaporated and was stored in −40°C until use. The water extracts of VU were further separated into polyphenol, anthocyanin-rich (pigment), and sugar/acid fractions by using ethyl acetate, acidic methanol, (MeOH), and 0.01 *N* HCl. 

### 2.2. Fractionation of *Vaccinium uliginosum L*. Extracts Using a C18 Sep-Pak Cartridge

To investigate the constituent of extract, a simple fractionation was performed using preconditioned C18 Sep-Pak cartridges to separate anthocyanins from nonanthocyanin phenolics. *Vaccinium uliginosum L*. dissolved in distilled water. Preconditioned by sequentially eluting with using ethyl acetate, acidic methanol, (MeOH), and 0.01 *N* HCl, respectively, with through the cartridges prepared total extract was loaded onto the catridges. Remove the fractions of sugar/acid with 0.01 *N* aqueous HCL. Cartridges were eluted with ethyl acetate (phenolic compounds layer) and phenolic compounds were then collected in a test tube. The absorbed anthocyanin was eluted from the cartridges with absolute MeOH with 0.1% (v/v) HCl and collected into test tubes. The solvents of fractions were removed using a rotary evaporator under reduced pressure at 20°C and 40°C, respectively. Therefore, we can obtain polyphenols and anthocyanins from VU, which is 1.8% and 4.1% of extract, respectively. Practice the fractionation of *Vaccinium uliginosum L*. extracts during the treatment, once a week. Such an oxidation of anthocyanin wasn't worth considering.

### 2.3. Animal

Four-week-old male NC/Nga mice were purchased from SLC (Shizuoka, Japan) and were housed in individually ventilated cages (IVC) at 23 ± 3°C in 55 ± 5% humidity with 12 h-12 h light-dark cycle (light on 7 : 30 am–7 : 30 pm) under SPF conditions in experimental period. Animals were maintained under constant environmental conditions and fed a standard laboratory diet (Jongang Lab Animal, Seoul, Korea) with water ad libitum. All experimental procedures were carried out in accordance with the protocol was approved by the Institutional Animal Care and Use Committee guideline of Kyung Hee University.

### 2.4. Induction of AD in the NC/Nga Mice

Chloro-2,4-dinitrobenzene (DNCB) (Sigma-Aldrich, St. Louis, MO, USA) was used to induce AD-like dermatitis in the negative control, positive control and treatment groups of NC/Nga mice [20]. Brief, dorsal hair of NC/Nga mice was removed by using electric shaver and hair removing cream containing thioglycolic acid repeatedly twice in a week. At the next day of last hair removal dorsal skin and right ear of NC/Nga mice were sensitized with 200 *μ*L of 1% DNCB mixture (1% DNCB dissolved in acetone and ethanol (2 : 3 v/v)). Four days later, 200 *μ*L of 1% DNCB mixture was treated once more for second sensitization. A week after first sensitization, the dorsal skin and right ear of NC/Nga mice were challenged with 150 *μ*L of 0.4% DNCB mixture (0.4% DNCB dissolved in acetone and olive oil (3 : 1 v/v)). Challenging with 0.4% DNCB mixture was repeated three times a week for 9 weeks.

### 2.5. Oral Administration of Mixture of Polyphenols and Anthocyanins

Induction of AD-like skin lesions in 9 weeks, the mice were divided into six groups according to the dose of mixture of polyphenols and anthocyanins: 0 (normal), 0 (negative control), 0 (positive control), poly+antho 2,56 + 7.31, 5.12 + 14.62, 10.25 + 29.25 *μ*g/kg·bw. Fractions were dissolved in 10% tween 80. For the normal and negative control groups, 10% tween 80 were administered instead of mixture of fractions. Also, the positive control group was treated with prednisolone (3 mg/kg·bw) dissolved in 10% tween 80. 

### 2.6. Measurement of Skin Severity and Ear Thickness

After the treatment of samples, the severity of dermatitis was assessed macroscopically in a blinded fashion using the following scoring procedure [21]. Briefly, dorsal and ear skin lesions were scored by the following criteria. The score was calculated as the sum of the individual score grades of the following five symptoms: dryness, lichenification (scaling), excoriation, erythema/edema, and erosion. For each skin symptom, individual score were graded as: 0 (no symptoms), 1 (mild), 2 (moderate), and 3 (severe). Skin severity score was evaluated by a single experienced person at 1 h before sample treatment. Ear thickness was measured three days a week using a thickness gauge (Mitutoyo Corporation, Tokyo, Japan) on the right ear of each mouse.

### 2.7. Measurement of Scratching Behavior Test

The total scratching behavior number was measured three times a week as described previously with slight modification [22]. Briefly, after oral administration of mixture or prednisolone, mice of each group were placed into a new clear plastic cage for 1 h of habituation. After habituation, the number of scratching episodes within 30 min was counted macroscopically [23]. A series of scratching movements made only with the hind paw was counted as one scratching episode. Each scratching episodes was scored from 0 to 4 : 0 (no scratching), 2 (scratching shorter than 1.5 s), and 4 (scratching longer than 1.5 s) [24]. The total scratching behavior number was calculated as sum of individual score.

### 2.8. Serum Immunoglobulin Analysis

Serum levels of immunoglobulin were determined using and enzyme-linked immunosorbent assay kits. The serum of IgE was determined by mouse IgE ELISA kit (Shibayagi, Japan) and that of IgG1, IgG2a were determined by mouse IgG1, IgG2a kits (Australia and New Zealand). The ELISA was performed in accordance with the manufacturer's instructions. After the last treatment, mice were anesthetized by diethyl ether and blood sample were collected from the inferior vena cava prior to sacrificing and was allowed to clot for 30 min at room temperature. The serum was prepared by centrifugation and stored at −70°C until use. The lower limits of detection of IgE, IgG1, and IgG2a were 1, 7.8, and 7.8 ng/mL respectively.

### 2.9. Splenic Cytokines Production

After the last treatment, mice were sacrificed by cervical dislocation and the spleens of each mouse was obtained. Seeded 24-well plate at concentration 1 × 10^6^ cells/well in RPMI 1640 medium, was supplemented with 10% FBS, 100 U/mL penicillin, and 50 *μ*g/mL streptomycin. Splenocytes were stimulated with 5 *μ*g/mL of Con A and incubated for 72 h. After incubation, medium from each well was aspirated and supernatant was collected by centrifugation and stored in −70°C until use. The levels of IL-4, IFN-*γ*, IL-12, and IL-13 in supernatant were measured by commercial ELISA kits (Australia and New Zealand, San Diego, Austin, and Texas and San Diego) according to each manufacturer's instructions. The lower limits of detection for IL-4, IFN-*γ*, IL-12, and IL-13 were 7.8, 7.8, 15.6, and 10 pg/mL, respectively.

### 2.10. Real-Time PCR Assay

At least five mice per group were used in each experiment. Total RNA was isolated from the skin tissues using TRIzol Reagent (Invitrogen) according to the manufacturer's protocol. After quantitative analysis of RNA were complete, reverse transcripted using Oligo dT (Promega), M-MLV reverse transcriptase (Promega), 10 mM dNTP mix, 5X cDNA symthesis buffer and RNase inhibitor. The amplification was carried out as follows: initial enzyme activation at 40 cycles of 42°C for 1 h, 94°C for 5 min and 4°C for 1 h. Preparations of cDNA were mixed with SYBR Green supermix (TaKaRa) mix and 5pM primer were analysed, performed on real-time PCR machine (TaKaRa Laboratories). According to the comparative Ct method, gene expression was normalized to the expression of the housekeeping gene *β*-actin [25]. The following primers were used for PCR reactions (5′–3′) (Table 1). Relative expression of each gene (fold-change to control) was calculated according to comparative Ct method using the formula: RQ = 2^−Ct^ with (ΔCt = Ct_Gene_ − Ct_18S_; Ct = ΔCt_Recovery_ − ΔCt_Control_) [26]. 

### 2.11. Histological Analysis

After the end of the experiment, dorsal and ear skins were fixed 10% formalin and embedded in paraffin wax. The sections were stained with H and E and Toluidine blue for detection of eosinophil and epidermal thickness counting and eosinophil or mast cell and degranulated mast cell.

### 2.12. Liver and Kidney Function Test

Serum levels of GPT and BUN activity were determined using and assay kits. The kit was performed in accordance with the manufacturer's instructions. 

### 2.13. Statistical Analysis

Data were expressed as the mean ± standard deviation (SD). Differences between groups were evaluated using Student's *t*-test. A *P*-value <0.05 was considered statistically significant.

## 3. Result 

### 3.1. Appraisal of AD-Like Symptoms Induced by Treatment of DNCB

**Figure 1:**
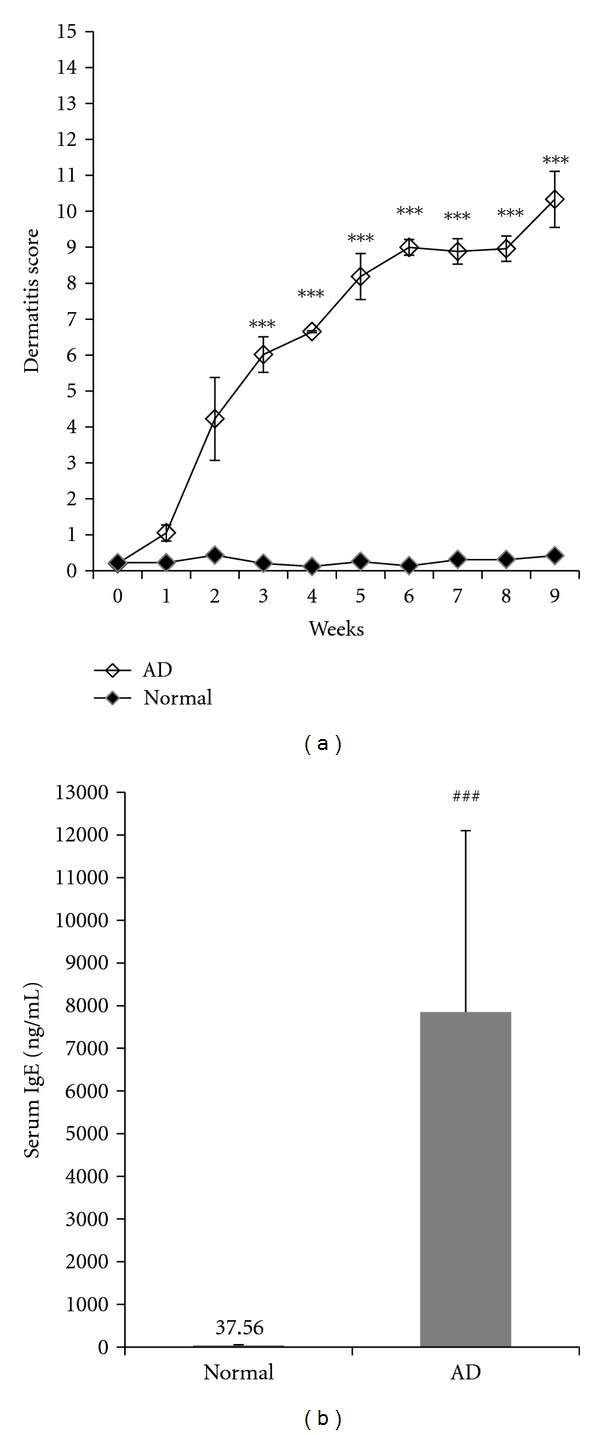
Evaluation of AD-like symptoms induced by treatment of DNCB. (a) Dermatitis score was assessed once a week for 9 weeks following induction of AD. Severity of dermatitis was evaluated as sum of the score for five clinical symptoms. (b) Serum was prepared a day after the last treatment with DNCB for measurement of serum total IgE level. Both the dermatitis score and serum total IgE levels were expressed as mean ± SD (NC group: *n* = 8, AD group: *n* = 40). ^###^ *P *< 0.001, significantly different from the normal group.

NC/Nga mice have been shown to develop AD-like skin lesions by repeated application of DNCB for 9 weeks. Normal group showed no physical signs of dermatitis whereas, skin induced AD group showed five symptoms of dryness, lich enification (scaling), excoriation, erythema/edema, and erosion on the ears and back. The dermatitis score of NC/Nga mice increased following the repeated application of DNCB. We obtained dermatitis scores ranging 11 to 12 after 9 weeks of DNCB treatment in atopic dermatitis group, and total serum IgE level of each mouse was measured. Total IgE level of AD group was 7849 ng/mL, which was much higher than for the normal group (37 ng/mL) (Figure 1). Experimental groups were divided into six groups based on these results. Thus, there was successful induced AD-like dermatitis. 

### 3.2. Allerviating Effect of the Mixture on Skin Severity

**Figure 2:**
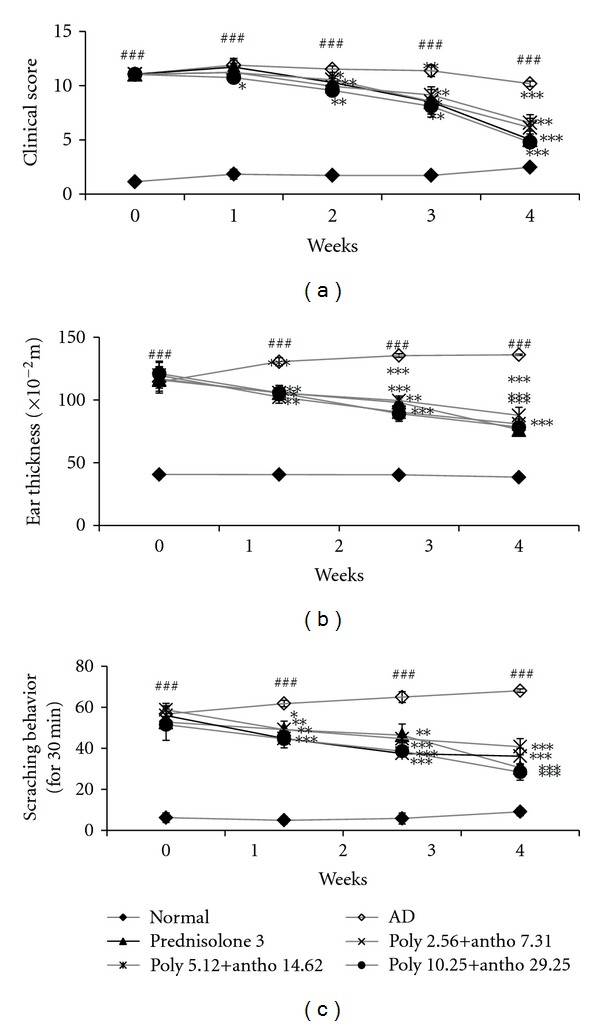
Alleviating effect of each dose of fractions on (a) dermatitis score, (b) Ear thickness and (c) scratching behavior. At the day 7, 14, 21, and 28 of treatment, clinical score and thickness of DNCB treated right ear of NC/Nga mice were measured. (a) Clinical skin score was evaluated as sum of the scores in five clinical symptoms. (b) Ear thickness was measured by dial thickness gauge. Both clinical skin score and ear thickness are expressed as mean ± SEM of five mice per group. Both clinical skin score and ear thickness are expressed as mean ± SEM of five mice per group. ^###^: *P* < 0.001 when compared with normal group and ^∗∗,∗∗∗^: *P* < 0.01, *P* < 0.001 when compared with 0.4% DNCB group. (c) The number of scratching behavior within 30 min was counted 1 h after each sample treatment, 3 times a week. Weekly data are expressed as mean ± SD of each scratching numbers of three days in a week. 0.01, ^###^ *P *<0.001, significantly different from the normal group. *P < 0.05, **P <0.01,***P < 0.001, significantly different from the 0.4% DNCB group.

**Figure 3:**
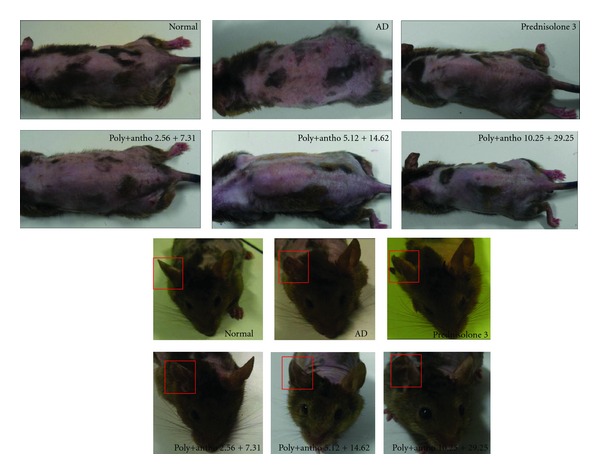
Clinical skin score and ear thickness. (a) Representative clinical features and (b) ear thickness of DNCB-induced NC/Nga mice in the untreated (normal) and treated groups with 2.56 + 7.31, 5.12 + 14.62, or 10.25 + 29.25 of polyphenols and anthocyanins and prednisolone (positive control).

NC/Nga mice were evaluated of AD-like symptoms induced by treatment of DNCB. We considered full development of AD at 9 weeks (the score >11). The NC/Nga mice developed AD induced by topical application of DNCB resulted in clinical signs and symptoms of dryness, lichenification (scaling), excoriation, erythema/edema, and erosion on the ears and back whereas normal controls did not exhibit any skin lesions (Figure 3). The scores were calculated from sums of the scores for five symptoms: with a score of 15 indicate the most severe state. The total clinical severity scores were significantly increased with time and reached a clinical score of high than induced group compared with the normal group. After of treatment for 4 weeks, six groups according to the dose of the mixture and positive control group significantly lowered the total clinical severity score compared to negative control group and the normal group showed no physical signs of dermatitis symptoms; this study shows that the positive control or the mixture treatment (poly+antho 2,56 + 7.31, 5.12 + 14.62, 10.25 + 29.25 *μ*g/kg·bw) groups suppressed AD-like lesions like that normal group (Figure 2(a)).

### 3.3. Effect of the Mixture on Ear Thickness

Ear thickness was significantly increased after inducing the atopic dermatitis by DNCB. To investigate the effect of the mixture on the severity of ear thickness in AD induced NC/Nga mice, the mixture of polyphenols and anthocyanins or prednisolone as a positive control were orally administrated daily for 30 days. Therefore, ear thickness was reduced by the mixture (poly+antho 2,56 + 7.31, 5.12 + 14.62, 10.25 + 29.25) groups and prednisolone (3 mg/kg·bw) treatment. The decrease rates of ear thickness in the mixture poly+antho 2,56 + 7.31, 5.12 + 14.62, 10.25 + 29.25 treated groups and positive control (predinisolone3 mg/kg·bw) group at 4 weeks were 24, 32, 35 and 34%, respectively (Figure 2(b)). After the last treatment, poly+antho 10.25 + 29.25 group exhibited decrease in ear thickness whereas positive control (predinisolone 3 mg/kg·bw) group did not. 

### 3.4. Inhibitory Effect of the Mixture Fractions in Scratching Behavior

Atopic dermatitis is characterized by severe itching and repeated scratching episodes. To estimate the involvement of scratching behavior, the number of scratching episode within 30 minutes was counted macroscopically 1 h after treatment, three times a week. The scratching number was counted as previously described with slight modification. After of the treatment with mixture and prednisolone groups showed suppressed scratching behavior in a time dependent manner. On the last week of treatment, only poly+antho 10.25 + 29.25 group showed significantly suppress in scratching number when compared with negative control group. Poly+antho 2.56 + 7.31, 5.12 + 14.62 groups showed significantly decrease on the second to third weeks, but the significance disappeared on the last week, whereas positive control group showed significantly reduced scratching episodes on last week (Figure 2(c)).

### 3.5. The Mixture Reduced the Infiltrations of Inflammatory Cells into DNCB-Induced Skin Lesions

**Figure 4:**
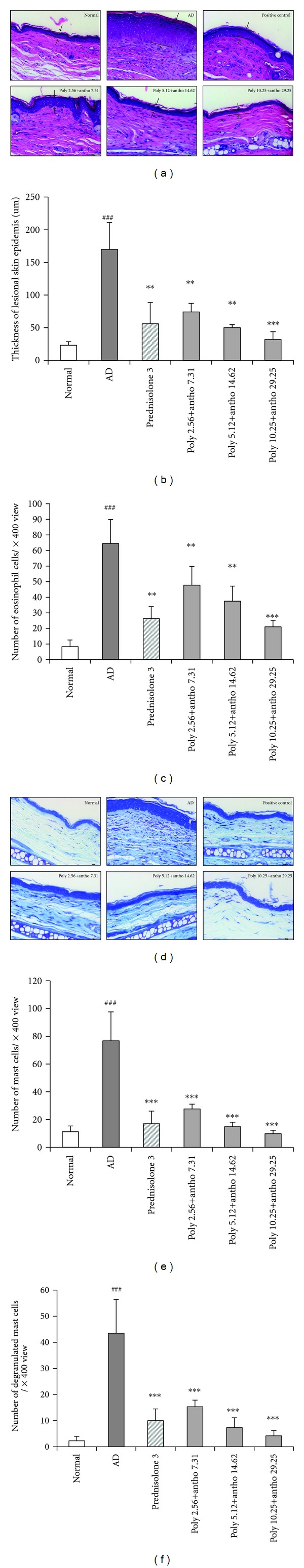
Histological changes by the mixture treatment. H&E or toluidine blue stained sections were observed under microscope at a magnification of ×400. (a) H&E stained lesional skin of normal, AD(negative control), prednisolone(positive control), poly+antho 2.56 + 7.31, 5.12 + 14.62, and 10.25 + 29.25 mg/mL treated groups (a) epidermal thickness and (b) infiltration of eosinophil was quantified as means in randomly selected four fields per mouse (d) Toluidine blue stained lesional skin of normal, AD(negative control), prednisolone(positive control), poly+antho 2.56 + 7.31, 5.12 + 14.62, and 10.25 + 29.25 mg/mL treated groups (e) infiltration of mast cell and (f) infiltration of degranulatd mast cell were quantified as means in randomly selected four fields per mouse. Scale bar = 100 *μ*m. Data are expressed as means ± SD of 5 mice per group. ^###^ *P *< 0.001, significantly different from the normal group. ***P < 0.001, significantly different from the AD group.

To investigate the effect of the mixture on histopathological AD like symptoms in NC/Nga mice, dorsal and ear skins were sectioned and stained with hematoxylin and eosin (H&E) (Figure 4(a)) or toluidine blue (Figure 4(d)) and observed under microscope at a magnification of ×400. The epidermal thickness of AD was 169 ± 41.2 while that of poly+antho 10.25 + 29.25 was 31.94 ± 12.04 *μ*m (*P *< 0.001) (Figure 4(b)). The infiltration of eosinophil (Figure 4(c)), mast cell, and degranulated mast cell were macroscopically counted in randomly selected four fields (area of ×400 view). We analyzed the swelling skin lesion and infiltration of mast cell (Figure 4(e)), degranulated mast cell (Figure 4(f)), and eosinophil. The number of infiltrated mast cell, degranulated mast cell and eosinophil were significantly decreased by the treatment the poly+antho 2,56 + 7.31, 5.12 + 14.62, 10.25 + 29.25 compared to control group. The mixture reduced the infiltrations of inflammatory cells in the lesional skin.

### 3.6. Effect of the Mixture on Serum Immunoglobulin

**Figure 5:**
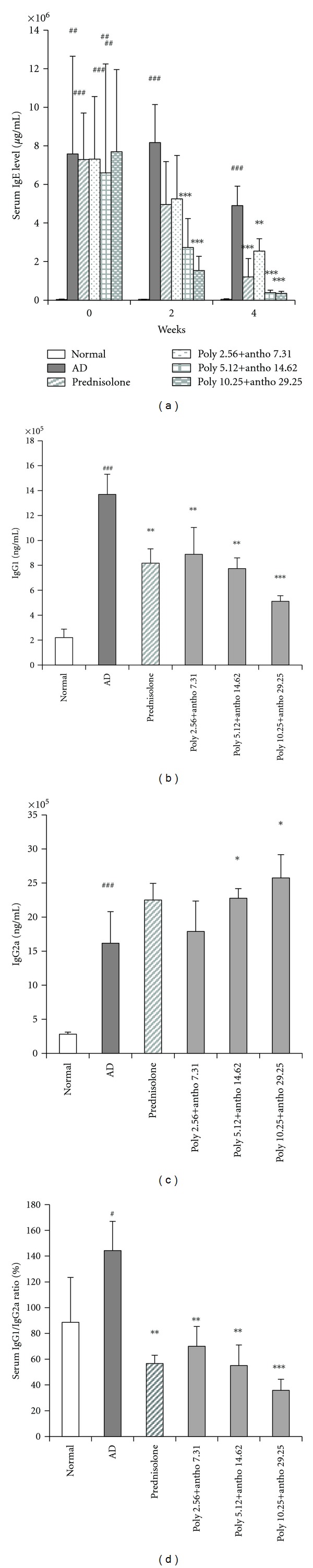
Levels of serum (a) IgE, (b) IgG1 (c) IgG2a and ratio of serum (d) IgG1/IgG2a. (a) On the 0, 2, and 4 weeks treatment, serum was collected after mice were sacrificed. Serum level of IgE was measured using ELISA kit. (b), (c) On day 28 of sample treatment, serum was collected after mice were sacrificed. Serum levels of (b) IgG1 and (c) IgG2a were measured using ELISA kit, (d) The IgG1/IgG2a ratio in serum was calculated as IgG1/IgG2a for each mice. Data are expressed as means ± SD of five mice per group. ^#^ *P *< 0.05, ^##^ *P *< 0.01,^ ###^ *P *< 0.001, significantly different from the normal group. *P < 0.05, **P < 0.01,***P < 0.001, significantly different from the AD group.

To investigate possible effects of the mixture in the serum IgE level, the application of DNCB significantly exhibited the serum IgE levels compared with the normal group. Sensitization with 0.4% DNCB highly increased the serum IgE level, and the treatment of the mixture of polyphenols and anthocyanins 2,56 + 7.31, 5.12 + 14.62, 10.25 + 29.25 or prednisolone decreased serum IgE levels with rates of 28, 58, 80 and 31% for 2 weeks. And IgE level with rates of 65, 94, 95 and 83% for 4 weeks respectively (Figure 5(a)). 

In serum of NC/Nga mice treated with DNCB, IgG1 level was elevated and IgG2a level was suppressed or not change. Poly+antho 5.12 + 14.62 group showed decrease serum IgG1 level as positive control group compared with negative control and poly+antho 10.25 + 29.25 group shows suppressed significantly than positive control groups (Figure 5(b)). Poly+antho 5.12 + 14.62 group increased serum IgG2a level as positive control group compared with negative control. Also, poly+antho 10.25 + 29.25 group elevated significantly then positive control (Figure 5(c)). In addition, induced AD be known to by unbalance of Th1/Th2 ratio, poly+antho 2,56 + 7.31, 5.12 + 14.62, 10.25 + 29.25 and positive control were decreased ratio of IgG1/IgG2a, which resulted 47, 58, 73 and 57% reduction in each groups respectively (Figure 5(d)). These results demonstrated that mixture fraction have immune regulative effects.

### 3.7. Effect of the Mixture in Splenic Cytokines Production

**Figure 6:**
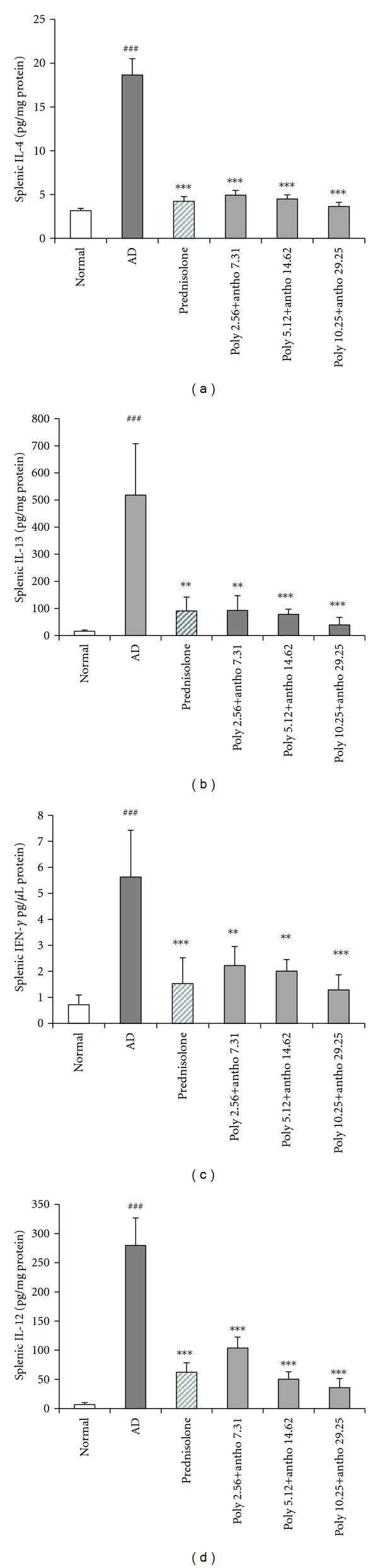
Effect of the mixture on splenic (a) IL-4, (b) IL-13, (c) IFN-*γ*, and (d) IL-12 production. At the day 28 of treatment, after mice were sacrificed and spleen of each mouse was obtained. Isolated splenocytes were stimulated by Con A and incubated for 72 h. The levels of (a) IL-4, (b) IL-13, (c) IFN-*γ*, and (d) IL-12 in supernatant were measured by ELISA kits. Data are expressed as means ± SD of five mice per group,^ ###^ *P *< 0.001, significantly different from the normal group. **P < 0.01,***P < 0.001, significantly different from the AD group.

To evaluate the statistical significance of the production of Th1 or Th2 cytokines by spleen, spleens were obtained from NC/Nga mice after scarification and isolated splenocytes were stimulated by Con A incubated for 72 h. The supernatant was collected after incubation and the levels of IL-4, IL-13 (as a Th2 cytokines), IFN-*γ* and IL-12 (as a Th1 cytokines) in supernatant were measured by ELISA kits. Treatment of the mixture or prednisolone dramatically suppressed both Th1 and Th2 cytokines production. The decreases rates of poly+antho 2,56 + 7.31, 5.12 + 14.62, 10.25 + 29.25 groups and positive control group for 73, 75, 80 and 77% for IL-4 level, 82, 84, 92 and 82% for IL-13 level 60, 64, 77 and 72% for IFN-*γ* level and 62, 82, 87 and 77% for IL-12 level respectively (Figure 6). In aggregate, poly+antho 5.12 + 14.62 group suppressed Th1 or Th2 cytokines levels as positive control group compared with negative control. Also, poly+antho 10.25 + 29.25 group suppressed than positive control group. Accompanied with the cytokines production of both poly+antho 2.56 + 7.31, 5.12 + 14.62, 10.25 + 29.25 groups and positive control group were similar to normal group.

### 3.8. The Mixture of Polyphenols and Anthocyanins Inhibit the Expression of Th1, Th2, and Th17 Cytokine Genes

**Figure 7:**
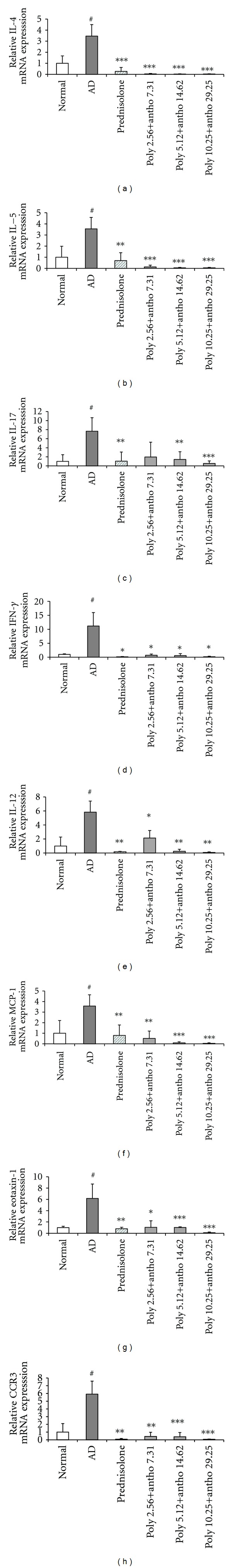
Effect of the mixture on the gene of IL-4, IL-5, IL-17, IFN-*γ*, IL-12, MCP-1, eotaxin-1, and CCR3 mRNA expression. At the last treatment, after mice were sacrificed and lesional skin tissues of each mice were obtained. Isolated RNA in the lesional skins. The levels of (a) IL-4, (b) IL-5, (c) IL-17, (d) IFN-*γ*, (e) IL-12, (f) MCP-1, (g) Eotaxin-1, and (h) CCR3 mRNA expressions were measured by real-time PCR machine. Data are expressed as means ± SD of five mice per group. ^#^ *P *< 0.05, significantly different from the normal group. *P < 0.05, **P < 0.01,***P < 0.001, significantly different from the AD group.

We analyzed the expression of genes implicated in atopic dermatitis in lesional skins of NC/Nga by quantitative PCR. To determine cytokines and chemokines production in the lesional skins, mRNA expression of Th1, Th2, and Th17 genes such as IL-4, IL-5, CCR3, eotaxin-1, IL-12, IFN-*γ*, MCP-1, and IL-17 were analyzed. We proved the effect of the mixture on the mRNA expression of cytokines in the lesional skins. The Th2, IL-4, and IL-5, are exhibited in acute stage of AD and the Th1, IL-12 and IFN-*γ*, is exhibited in chronic stage of AD. In this study, the lesional skin showed much higher expression levels of Th1, Th2, and Th17 genes, especially IL-4, IL-5, IL-17, and IFN-*γ* mRNA increased than normal group (Figure 7). In the result, the suppressed expression of IL-4, IL-5, CCR3, eotaxin-1, IL-12, IFN-*γ*, MCP-1, and IL-17 by treatment with polyphenols and anthocyanins compared to the positive control. Especially, the mixture poly+antho 2.56 + 7.31, 5.12 + 14.62, 10.25 + 29.25 groups were exhibited as similar to normal group.

### 3.9. Liver and Kidney Function Tests

Glutamate pyruvate transaminase (GPT) activity was measured to evaluate the hepatic dysfunction. Blood urea nitrogen (BUN) test is a measure of the amount of nitrogen in the blood in the form of urea for measured to assess kidney dysfunction. There were determine with an ALT/SGPT and BUN ELISA kit in serum. There was no increased serum of GPT and BUN levels in the treatment the mixture and prednisolone (Table 2). Thus, the mixture of polyphenols and anthocyanins has not been toxicity for liver and kidney.

## 4. Discussion

**Figure 8:**
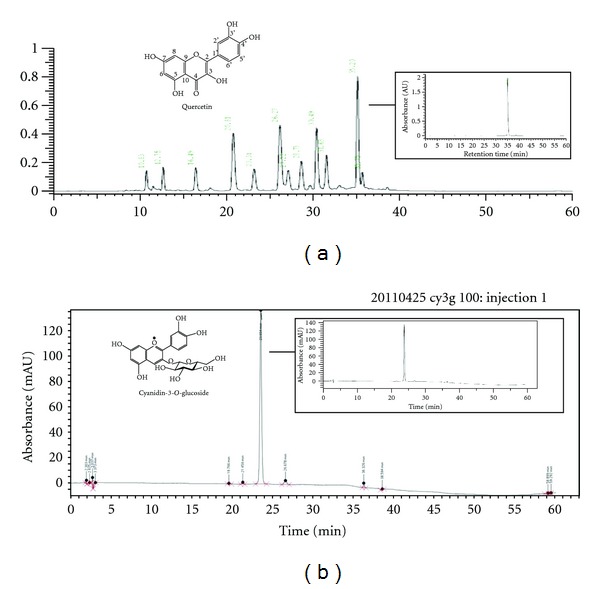
The HPLC chromatogram of the polyphenol and anthocyanin from VU Identification of (a) quercetin and (b) cyanidin-3-o-glucoside in polyphenols and anthocyanins, respectively, and chemical structure of quercetin and cyanidin-3-o-glucoside.

AD is defined as a chronic inflammatory skin condition characterized by intense pruritus and a series of exacerbations and remissions [27]. The incidence of AD is growing, especially in industrialized countries [28]. Research about the cause of AD is progressing and new treatments are being developed, but these agents have severe side effects, which limit their clinical applications [29]. Recently, treatments for AD lesions include the use of natural agents such as licorice, green tea, soybeans, acai berries, turmeric, and pomegranate [30]. The fruit of VU has been reported to contain polyphenol, anthocyanin, and sugar/acid. To investigate the constituents of the extract, a simple fractionation was performed using preconditioned C18 Sep-Pak cartridges to separate anthocyanins from nonanthocyanin phenolics. Polyphenols and anthocyanins, in particular, are known to be kinds of flavonoids. Flavonoids have been recognized to have antioxidant, antibacterial, and antiviral ability and to possess antiinflammatory, antiangiogenic, analgesic, hepatoprotective, cytostatic, apoptotic, estrogenic, or antiestrogenic properties, as well as antiallergic effects [31, 32]. In this study, we isolate the polyphenol and anthocyanin from VU using HPLC, and the compound was identified as quercetin and cyanidin-3-o-glucoside by NMR spectroscopy, respectively (Figure 8). In our *in vitro* assay using isolated mice splenocytes, polyphenols and anthocyanins fraction effectively inhibited the production of IL-4 (major of Th2 cytokine) which plays an important role in atopic dermatitis disease (data not known). Thus, in this study, we investigated how mixture fractions, such as polyphenols and anthocyanins derived from VU, alleviate DNCB-induced AD in an AD mouse model. We examined NC/Nga mice that showed AD-like skin lesions with aging as a possible mouse model for AD [33] and maintained them under specific pathogen-free (SPF) conditions. After negative and positive control groups and treatment with the mixture groups induced DNCB, therefore NC/Nga mice with AD lesions show clinical symptoms, including dryness, lichenification (scaling), excoriation, erythema/edema, and erosion the results show that the control group also developed symptoms, whereas the development of AD lesions decreased in the control group. The dermal infiltration of inflammatory cells is an important histopathological feature of atopic dermatitis [34]. Activated mast cells release a variety of biologically active substances that play important roles in allergic reactions, such as AD [35]. Inflammatory factors decreased after oral administration of the mixture. Treatment with the mixture alleviated histopathological symptoms in dorsal and ear skin, including infiltration of eosinophils, mast cells, and degranulated mast cells. In the skin, IgE can be traced to Fc receptors on cutaneous mast cells and on antigen-presenting dendritic cells (DCs). Suppression of degranulated mast cells was indirectly correlated with reduced histamine and the inhibited infiltration of eosinophils was correlated with decreased IL-4 and IL-13 production. This is in line with a recent study that suggested such Th2 cytokines were implied in eosinophil recruitment [36]. The lesion showed high serum IgE levels by the switching of B cells from IgM and IgE through the mediation of IL-4 upregulation [37, 38]. In fact, effect of AD (atopic dermatitis) was a good result in vivo system than *in-vitro* system. In my guess, the reason is different to treatment a period. The mixture or prednisolone significantly reduced total serum IgE levels. AD is developed to successive phase: acute AD skin lesions are characterized by the local expression of Th2 cytokines whereas chronic AD skin lesions are characterized by mixed expression of both Th2 and Th1 cytokines [39, 40]. Recent studies suggest a key role of the Th1-type cytokine IFN-*γ* in the chronicity of AD lesions [41–43]. Chronic phase of AD were compared with health state via production of IFN-*γ* and IL-12 by IDEC [44]. The MCP-1 mRNA signal was absent in baseline conditions [45] and potential attracts monocytes and dendritic cells *in vivo*, [46] but it can induce migration of both Th1 and Th2 cells [39]. Previous studies have shown that the mixture groups can suppress activation of IL-12, IFN-*γ*, and MCP-1 mRNA in AD-like skin lesions. In atopic dermatitis Th2-cells play an important role in the initial phase of inflammatory reactions whereas in later stages Th1-cells can be detected in greater numbers [47]. Repeated treatment with DNCB released both Th1 and Th2 cytokines by activating helper T cells. We discovered that treatment with the mixture of fractions from VU significantly decreased both Th1 and Th2 cytokines in spleens. Hence, we believe the mixture has an effect on both acute and chronic AD lesions. The important thing is the lesion due to the Th1/Th2 imbalance skewed to Th2, which plays an important role in the pathology of AD [48]. The important thing is the segregation of IgG2a and IgG1 immunoglobulin isotypes as markers for Th1 and Th2 lymphocytes, respectively, for each ratio [49]. We were show the alleviate effect of Th1/Th2 balance through the IgG2a and IgG1 ratio. Oral administration of the mixture for 4 weeks suppressed IgG1 levels and increased IgG2a levels in a dose-dependent manner. The evaluated IgG1/IgG2a ratio showed the mixture also significantly reduced the Th2/Th1 ratio. Itching is one of the major features of AD and scratching causes lesions to relapse [50, 51]. Therefore, effective regulation of itching and scratching is important and required for AD patients using real-time PCR analysis the mixture inhibited expression of IL-4 mRNA, which is the main Th2 cytokine that activates B cells, eosinophils, and mast cells. Interleukin-5 mRNA is also found in activated eosinophils in tissues from AD patients [52–54]. Eotaxin-1 (CCL11) mRNA is produced by epithelial and endothelial cells, with CCR3 being predominately localized to eosinophils [55]. The expression of IL-5, CCR3, and Eotaxin-1 (CCL11) mRNA were significantly decreased as a result of treatment with the mixture. In addition, a subset of IL-17-inducing Th cells (Th17) was recently identified and shown to play an important role in tissue inflammation [56, 57]. Recently, the Th1/Th2 paradigm in autoimmunity and allergic reactions has been revisited, including the role for a new population of IL-17-producing Th cells (Th17) [46]; Ingeminated application DNCB especially increased mRNA expression of Th1 and Th2related genes. In addition, previous study reports an increase in Th17 cells in the inflamed skin of AD patients [58]. However, their role in AD is still unclear. The potential involvement of Th17 T-cells in AD has been suggested [46]. Elevated IL-17 T cells have been found in tissue sections of AD lesions relative to normal skin [45]. Our previous work on chronic AD-like skin lesions also proved increased IL-17 expression compared with normal and treatment groups were dwindled expression of IL-17 mRNA. We found that IL-17 was also associated with acute and chronic AD lesions. Ultimately, the mixture of polyphenols and anthocyanins has an effect of clinical histological and immunological gene expression on AD-like skin lesions. Nevertheless the mixture is natural and has the strong effect of AD-like skin lesions. The advantage of oral administration of the mixture is that it appeared not to induce liver and kidney dysfunction. Therefore, the mixture is a good candidate for anti-AD drugs of complementary and alternative medicine.

## 5. Conclusion

The mixture of polyphenols and anthocyanins has been successfully applied in treating AD-like lesions, inhibiting inflammation of the lesion skin, correcting the Th1/Th2 balance, and reducing IL-17. This treatment is justified to elucidate the interplay between these Th cell subsets in acute and chronic AD [59] in DNCB-treated NC/Nga mice. Topical application of the mixture may therefore be a novel approach to the treatment of atopic dermatitis. 

## Figures and Tables

**Table 1:** Primers of real time PCR.

Gene	Quantification method	Sequence (5′-3′)	Tm (°C)
*β*-actin	Forward primer	CCCAACTTGATGTATGAAGG	55
	Reverse primer	TTGTGTAAGGTAAGGTGTGC	55

Eotaxin-1/CCL11	Forward primer	CACCCTGAAAGCCATAGTGT	57
	Reverse primer	TGTGTACCTGGGAAATTAG	53

IL-4	Forward primer	GTCTGCTGTGGCATATTCTG	57
	Reverse primer	GGCATTTCTCATTCAGATTC	53

IFN-gamma	Forward primer	CTCTGAGACAATGAACGCTACACACT	61
	Reverse primer	TGGCAGTAACAGCCAGAAACAG	60

IL-5	Forward primer	GGCTACACAGAGAAACCCTGT	59
	Reverse primer	CATGCATACACAGGTAGTTCA	55

CCR4	Forward primer	TCGCCTTGTTTCAGTCAGG	57
	Reverse primer	CTTGCCATGGTCTTGGTTTT	55

CCR3	Forward primer	CCCGTACAACCTGGTTCTCC	61
	Reverse primer	AAAGAGCCGAAGGTGTTTCC	57

MCP-1	Forward primer	TTAAGGCATCACAGTCCGAG	57
	Reverse primer	TGAATGTGAAGTTGACCCGT	55

IL-17	Forward primer	AAGGCAGCAGCGATCATCC	59
	Reverse primer	GGAACGGTTGAGGTAGTCTGAG	61

**Table 2:** Liver and kidney function tests. GPT and BUN activities were measured to assess hepatic and nephrotoxic dysfunction. (a) GPT and (b) BUN were measured by ELISA kit. Data are expressed as means ± SD of five mice per group.

Group (mg/mL)	IU/L	mg/dL
	ALT/SGPT	BUN
Normal	29.23 ± 4.85	23.42 ± 3.09
AD	24.23 ± 2.77	21.32 ± 2.52
Prednisolone 3	37.45 ± 9.65	23.05 ± 2.15
Polyphenol + anthocyanin		
2.56 + 7.31	29.01 ± 7.53	23.27 ± 2.55
5.12 + 14.62	29.55 ± 9.91	24.81 ± 3.42
10.25 + 29.25	30.68 ± 3.89	24.96 ± 1.99

## Abbreviations

AD: Atopic dermatitis
DNCB: 2,4-dinitrochlorobenzene
Poly+antho: polyphenol + anthocyanin
VU: *Vaccinium uliginosum L*.
H&E stain: Hematoxylin-eosin stain
IL: Interleukin
CCL: Chemokine (C-C motif) ligand
CCR: Chemokine (C-C motif) receptor
MCP-1: Monocyte chemoattractant protein 1
Ig: Immunoglobulin.
